# Tumoral Melanosis Arising on a Mycosis Fungoides Plaque

**DOI:** 10.4274/balkanmedj.2018.0936

**Published:** 2018-11-15

**Authors:** Hatice Gamze Demirdağ, Bengü Nisa Akay, Ayça Kırmızı, Aylin Okçu Heper

**Affiliations:** 1 Department of Dermatology, University of Health Sciences, Dr. Abdurrahman Yurtaslan Ankara Oncology Training and Research Hospital, Ankara, Turkey; 2 Department of Dermatology, Ankara University School of Medicine, Ankara, Turkey; 3 Department of Pathology, Ankara University School of Medicine, Ankara, Turkey

To the Editor,

Tumoral melanosis is a rare histopathological phenomenon characterized by confuent dermal nodular aggregates of heavily melanized polygonal cells consistent with melanophages. The initial occurrence of tumoral melanosis always raises the suspicion of a regressed melanocytic proliferation, especially a melanoma. Regression can be recognized by the presence of dermal fibrosis, vascular proliferation, and usually a band-like infiltrate of lymphocytes and melanophages in variable numbers. However, these changes are non-specific, and similar findings can be seen in regression of pigmented epithelial lesions, such as basal cell carcinoma, Bowen’s disease, solar lentigo, and rarely, in mycosis fungoides (1,2). We present a case of tumoral melanosis which arose on a plaque of mycosis fungoides and discuss the possible underlying pathological mechanisms.

A 69-year-old male presented with a nodule on his right arm. He had folliculotropic mycosis fungoides and was being followed-up with 3 months interval. During the physical examination, a 4×4 mm, flat, roundish, blue-gray pigmented lesion on a 3×4 cm erythematous plaque, which had not been present at the previous 3-month follow-up, was detected (Figure 1a). Dermatoscopic examination revealed irregular blue clods with shiny white lines on a grayish-brownish structureless area surrounded by dotted vessels (Figure 1b). Histopathology of this pigmented lesion showed acanthotic epidermis with melanin pigmentation of basal keratinocytes and papillary dermal nodular accumulation of melanophages and lymphocytes, which were consistent with tumoral melanosis. Melanin pigmentation faded away after melanin decolorization, and melanophages were diffusely positive for CD68 immunohistochemically (Figure 1c, 1d). Another biopsy adjacent to the tumoral melanosis showed the plaque stage of mycosis fungoides histopathologically (Figure 1e). Since there was possibility of a totally regressed melanoma, sentinel lymph node biopsy and positron emission tomography imaging were performed. There was no evidence of metastatic melanoma. Written informed consent was obtained from the patient.

Tumoral melanosis is usually considered as a manifestation of fully regressed melanoma. However, this finding does not seem to be pathognomonic for melanoma (1). The nature of the underlying lesion is not always clear. The present case was under close follow-up for his mycosis fungoides; physical examination was performed at every visit, and if a melanoma had been present, it should not have been overlooked.

The distinctive feature of carcinogenesis is macrophage-mediated inflammation, often caused by pathogens or a result of autoimmunity and inflammatory conditions of unknown origin. The Th1-macrophage response controls the early phases of tumor development (3). The most common types of cutaneous T-cell lymphoma are mycosis fungoides and Sezary syndrome. Although the pathogenesis of cutaneous T-cell lymphoma is not yet very well understood, a variety of cytokines/chemokines are reported to take part in disease development. Dermal macrophages are the possible sources of these cytokines/chemokines. Tumor-associated macrophages usually express the M2 phenotype, which has been associated with tumor growth induction and poor prognosis. In human studies on cancer, macrophages have predominantly been identified by immunohistochemistry applying antibodies against CD68, which is a classical macrophage marker. M2 macrophages express high levels of CD163, a hemoglobin-scavenger receptor, and can be used to discriminate between M1 and M2 macrophages (3). In a study, CD163+ cells were detected in the invasive margin of the tumor in cutaneous T-cell lymphoma (4). Ueno et al. (5) reported massive infiltration of CD68+ and CD163+ M2 macrophages in a case with tumoral melanosis. They suggested that an immunosuppressive microenvironment created by M2 macrophages may participate in the formation of tumoral melanosis. In our case, the possible underlying pathological mechanism seems to be the interactions between lymphocytes and macrophages leading to an increased number of M2 macrophages and accumulation of pigmented melanophages without dermal melanocytes.

In conclusion, wide spectrum of skin lesions other than melanoma should also be considered in the development of tumoral melanosis.

## Figures and Tables

**Figure 1:**
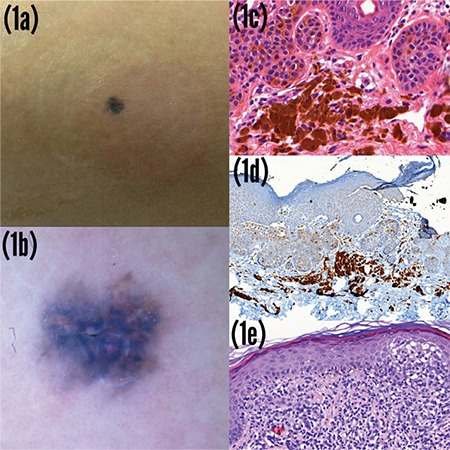
a-e. A 4×4 mm solitary, flat, roundish, blue-gray pigmented lesion on a 3×4 cm erythematous plaque (a), Dermatoscopy shows large blue clods with shiny white lines on a grayish-brownish structureless area surrounded by dotted vessels (b), Acanthotic epidermis with melanin pigmentation of basal keratinocytes and papillary dermal nodular accumulation of melanophages and lymphocytes, consistent with tumoral melanosis (hematoxylin and eosin, X40) (c), Dermal melanophages were diffusely positive with CD68 immunohistochemistry (CD68, X20) (d), Epidermotropism of atypical lymphocytes and plaque formation of atypical lymphocytes in the papillary dermis, consistent with mycosis fungoides (hematoxylin and eosin, X30) (e).
